# Supplementary material for: Profiling Novel Alternative Splicing within Multiple Tissues Provides Useful Insights into Porcine Genome Annotation
Source: Genes (Basel). 2020 Nov 26;11(12):1405. doi: 10.3390/genes11121405 (PMC7760890; doi:10.3390/genes11121405)
Supplement: Supplementary file 1 [file genes-11-01405-s001.zip › genes-1018581-suppl/supplementary figures.pdf]

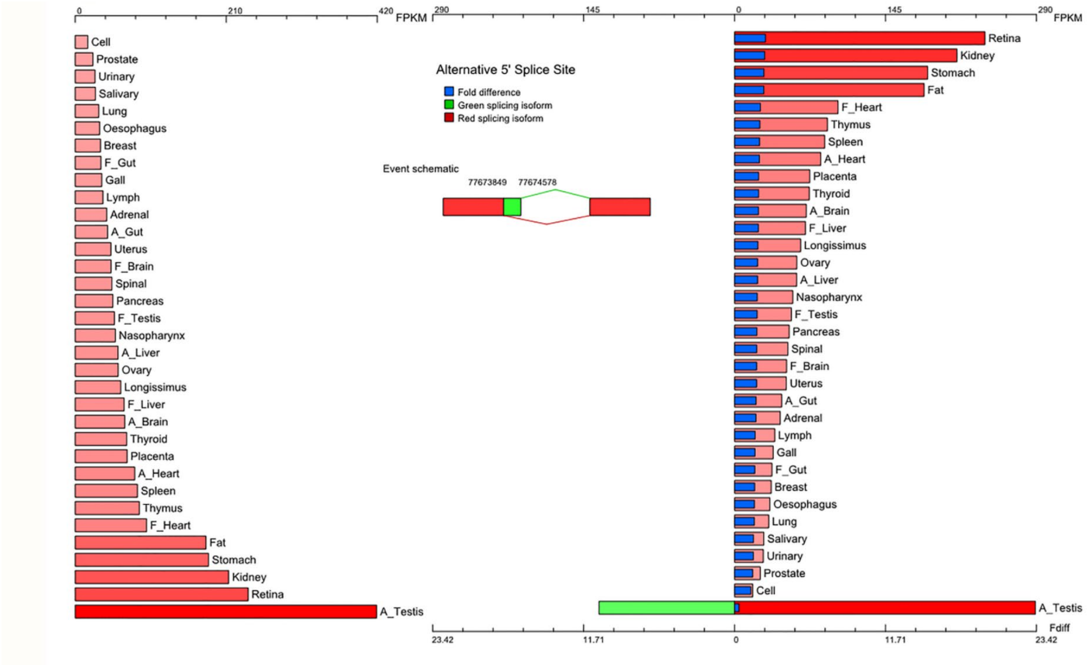

Figure S1. The specific AS events of the GPX4 gene in the testis of adult pigs. Bar plots on the left-side show the expression levels of GPX4 gene for each tissue. Bar plots on the right-side show expression levels of GPX4 transcript for each tissue. Green and red splicing isoforms represent the novel and canonical splicing, respectively. The blue bar represents fold difference (expression levels of canonical splicing/expression levels of gene).

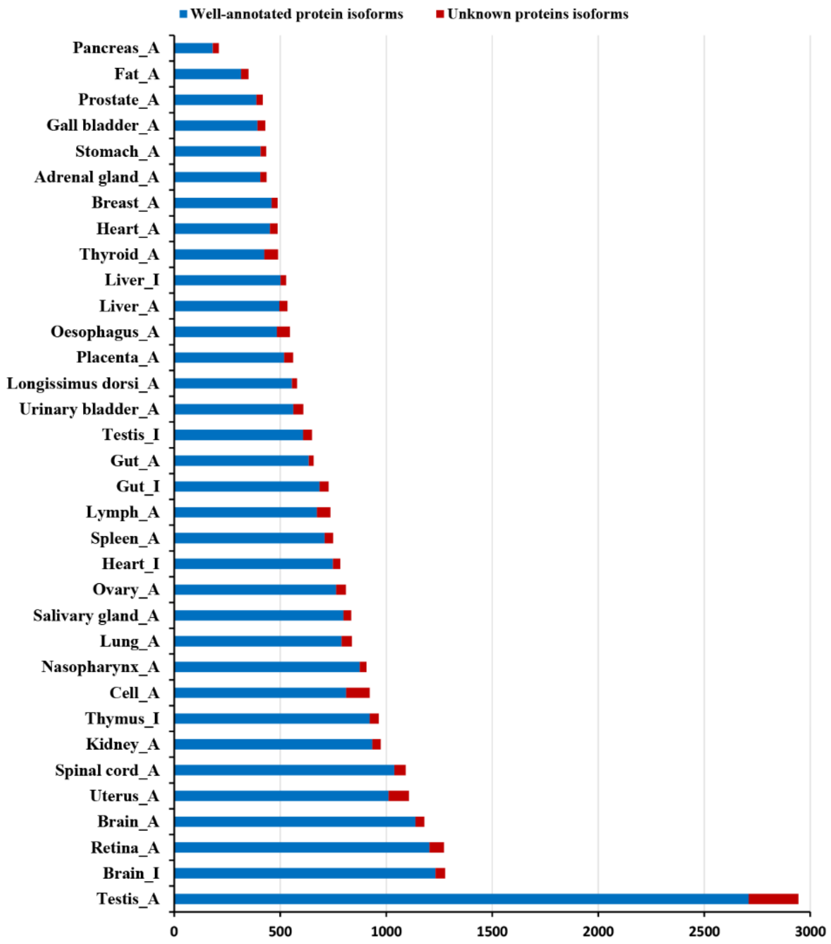

Figure S2. Numbers of tissue-enriched isoforms for known and unknown proteins. Blue bars and red bars represent the number of isoforms of well annotated proteins and unknown proteins, independently.

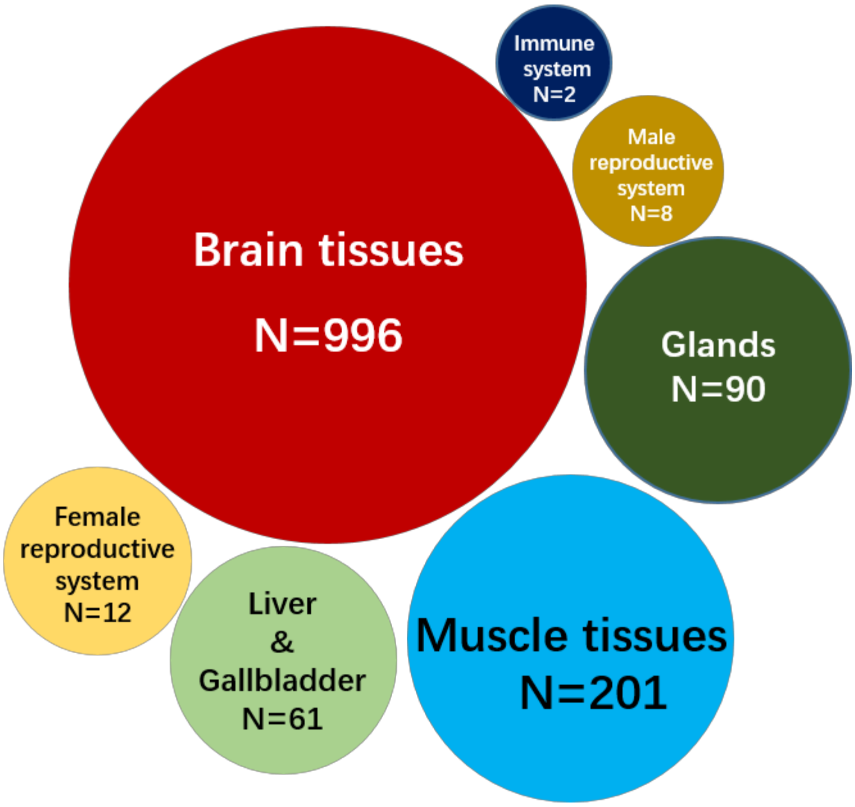

Figure S3. Numbers of group-enriched isoforms in different tissue groups
